# Contrast-free detection of myocardial fibrosis in hypertrophic cardiomyopathy patients with diffusion-weighted cardiovascular magnetic resonance

**DOI:** 10.1186/s12968-015-0214-1

**Published:** 2015-12-02

**Authors:** Christopher Nguyen, Minjie Lu, Zhaoyang Fan, Xiaoming Bi, Peter Kellman, Shihua Zhao, Debiao Li

**Affiliations:** Biomedical Imaging Research Institute, Cedars-Sinai Medical Center, Los Angeles, CA USA; Department of Bioengineering, University of California Los Angeles, Los Angeles, CA USA; State Key Laboratory of Cardiovascular Disease, Fuwai Hospital, Beijing, China; National Center for Cardiovascular Diseases, Chinese Academy of Medical Sciences and Peking Union Medical College, Fuwai Hospital, Beijing, China; MR R&D, Siemens Healthcare, Los Angeles, CA USA; National Heart, Lung, and Blood Institute, National Institutes of Health, Bethesda, MD USA

**Keywords:** Hypertrophic cardiomyopathy, HCM, Diffusion-weighting, Cardiovascular magnetic resonance, Extracellular volume mapping, ECV

## Abstract

**Backgrounds:**

Previous studies have shown that diffusion-weighted cardiovascular magnetic resonance (DW-CMR) is highly sensitive to replacement fibrosis of chronic myocardial infarction. Despite this sensitivity to myocardial infarction, DW-CMR has not been established as a method to detect diffuse myocardial fibrosis. We propose the application of a recently developed DW-CMR technique to detect diffuse myocardial fibrosis in hypertrophic cardiomyopathy (HCM) patients and compare its performance with established CMR techniques.

**Methods:**

HCM patients (*N* = 23) were recruited and scanned with the following protocol: standard morphological localizers, DW-CMR, extracellular volume (ECV) CMR, and late gadolinium enhanced (LGE) imaging for reference. Apparent diffusion coefficient (ADC) and ECV maps were segmented into 6 American Heart Association (AHA) segments. Positive regions for myocardial fibrosis were defined as: ADC > 2.0 μm^2^/ms and ECV > 30 %. Fibrotic and non-fibrotic mean ADC and ECV values were compared as well as ADC-derived and ECV-derived fibrosis burden. In addition, fibrosis regional detection was compared between ADC and ECV calculating sensitivity, specificity, positive predictive value (PPV), and negative predictive value (NPV) using ECV as the gold-standard reference.

**Results:**

ADC (2.4 ± 0.2 μm^2^/ms) of fibrotic regions (ADC > 2.0 μm^2^/ms) was significantly (*p* < 0.01) higher than ADC (1.5 ± 0.2 μm^2^/ms) of non-fibrotic regions. Similarly, ECV (35 ± 4 %) of fibrotic regions (ECV > 30 %) was significantly (*p* < 0.01) higher than ECV (26 ± 2 %) of non-fibrotic regions. In fibrotic regions defined by ECV, ADC (2.2 ± 0.3 μm^2^/ms) was again significantly (*p* < 0.05) higher than ADC (1.6 ± 0.3 μm^2^/ms) of non-fibrotic regions. In fibrotic regions defined by ADC criterion, ECV (34 ± 5 %) was significantly (*p* < 0.01) higher than ECV (28 ± 3 %) in non-fibrotic regions. ADC-derived and ECV-derived fibrosis burdens were in substantial agreement (intra-class correlation = 0.83). Regional detection between ADC and ECV of diffuse fibrosis yielded substantial agreement (κ = 0.66) with high sensitivity, specificity, PPV, NPV, and accuracy (0.80, 0.85, 0.81, 0.85, and 0.83, respectively).

**Conclusion:**

DW-CMR is sensitive to diffuse myocardial fibrosis and is capable of characterizing the extent of fibrosis in HCM patients.

## Backgrounds

Detecting and characterizing interstitial diffuse myocardial fibrosis has significant prognostic value for cardiovascular disease patients [1–3]. Current cardiovascular magnetic resonance (CMR) methods to characterize diffuse myocardial fibrosis include late gadolinium enhanced imaging (LGE) [3, 4], post contrast T1 mapping [5–7], and extracellular volume (ECV) mapping [7–9]. The latter two techniques provide quantitative measures (T1 and ECV values) that can further characterize the degree of fibrosis. However, these conventional techniques require the use of contrast and are contraindicative in patients with renal insufficiency. Contrast-free quantitative CMR techniques such as native T1 mapping [10], diffusion imaging [11–13], T1ρ imaging [14], and Creatine chemical-exchange imaging [15] have shown promise in detecting replacement myocardial fibrosis (i.e. scar) in chronic myocardial infarction (MI). Of these techniques, native T1 mapping [16] and diffusion imaging [17, 18] have demonstrated additional sensitivity to diffuse myocardial fibrosis.

Currently, in vivo diffusion tensor CMR (DT-CMR) has been shown to be sensitive to the presence of myocyte disarray [19] and abnormal myocardial sheetlet mechanics [20] in hypertrophic cardiomyopathy (HCM). However, simple in vivo diffusion-weighted CMR (DW-CMR), which requires less than half the measurements of DT-CMR, may also have potential in identifying diffuse myocardial fibrosis in HCM. Pop, et al. and Abdullah, et al. demonstrated with histological validation that ex vivo DW-CMR has the ability to characterize the border-zone fibrosis region of chronic MI scar [14] and diffuse myocardial fibrosis in failing hearts [18], respectively. DW-CMR was able to not only detect diffuse interstitial fibrosis, but also quantify the degree of fibrosis showing strong correlation between apparent diffusion coefficient (ADC) and the percent fibrosis. In both studies, the minimum amount of fibrosis to cause a significant change in ADC was 20 % of fibrosis.

Therefore, it is expected that in vivo estimates of ADC should also be sensitive to both diffuse and replacement myocardial fibrosis if a sufficient amount of fibrosis is present (≥20 %).We propose the application of a recently developed DW-CMR technique [21] to detect myocardial fibrosis in HCM patients and compare its performance with histologically validated in-vivo contrast-enhanced CMR techniques such as ECV and LGE.

## Methods

### Patient recruitment

All patients (*N* = 23) gave informed consent to the protocol, which was approved by Institutional Review Board of Fuwai Hospital. The HCM was diagnosed (or confirmed) by the presence of a non-dilated and hypertrophied LV on echocardiography or CMR (maximal wall thickness ≥15 mm in adult index patients or ≥13 mm in adult relatives of HCM patients) in the absence of another disease that could account for the hypertrophy [22]. Patients who were known to have coronary artery disease, aortic stenosis, amyloidosis, systemic hypertension, or contraindications to CMR imaging were not included. Patients with previous septal ablation or myectomy were excluded. Among the 23 patients, 19 are asymmentrical type including 14 obstructive HCM and 5 non-obstructive HCM, the remaining 4 are apical HCM. Regarding to the LV functional parameters, the mean maximal wall thickness, LV mass, LVEF, diastolic and systolic volumes are 22.8 ± 7.6 mm, 130 ± 52 g, 65.4 ± 69 %, 66.2 ± 16.1 ml/m^2 and 23.1 ± 7.5 ml/m^2, respectively. Patient characteristics are displayed in Table 1.

**Table 1:** HCM Patient Characteristics

	Patients (*n* = 23)
Ages(years, mean ± SD)	50.0 ± 17.5(29,59)
Gender (male/female)	14/9
Body mass index(kg/m^2^)	22.3 ± 2.8
Systolic Blood pressure(mmHg)	114 ± 12
Systolic Blood pressure (mmHg)	78 ± 9
Family History of HCM(n, %)	8(34.8)

Data presented are n (%) for categorical variables and median ± standard deviation for continuous variables

### MRI protocol

All patients were scanned on at 1.5 T clinical scanner (MAGNETOM Avanto, Siemens Healthcare, Erlangen, Germany) with the following protocol: standard morphological localizers, CINE, DW-CMR (one b0 image, three orthogonal diffusion directions, b = 350 s/mm^2, second order motion compensation diffusion-prepared bSSFP) [21], ECV-CMR (pre/post contrast T1 mapping modified look locker imaging) [8, 9], and LGE (Table 2). Diffusion encoding of the DW-CMR was played out during the most quiescent period of the cardiac cycle identified by standard CINE imaging (typically end systole or end diastole) and the exhalation respiratory phase to match ECV-CMR and LGE breath-hold positions. From previous CINE imaging of 3 HCM patients, we determined that the quiescent period duration ranged from 50 to 80 ms. Therefore, we tailored the DW-CMR sequence to have a shorter diffusion preparation time (TE_prep_ = 80 ms) compared with the diffusion preparation time used in healthy volunteers (TE_prep_ = 115 ms) [21]. This also increased SNR by 2-fold to offset the loss in singal-to-noise ratio of reducing slice thickness from 10 mm to 8 mm to match DW-CMR with ECV-CMR and LGE. ECV-CMR and LGE were always acquired during end diastole. DW-CMR was acquired at four contiguous short-axis slices centered about the mid LV due to its 3D acquisition. For ECV-CMR and LGE with 2D acquisition, three short-axis slices (base, mid, and apex) were acquired. Because of the large LV mass of HCM patients (typically 10 cm long-axis length), only the mid short axis slice was consistently matched across all scans.

**Table 2:** CMR Parameters

	Diffusion CMR	ECV CMR	LGE CMR
Spatial Resolution	1.6 × 1.6 × 8 mm^3^	2.1 × 1.9 × 6 mm^3^	1.5 × 1.5 × 6 mm^3^
TR	4.1 ms	2.4 ms	3.3 ms
TE	2.0 ms	1.1 ms	1.4 ms
Flip Angle	110°	35°	25°
Shots	4	1	6
Magnetization Prep Timing	TE_prep_ = 80 ms	TI_min_ = 110 ms	TI = 300 ms
		TI_increment_ = 80 ms	
Respiratory Mode	Free Breathing	Breath Hold	Breath Hold
Scan Time	5 to 7 min	6 min	6 min

### Image analysis

ADC maps were calculated for each of the three diffusion directions (ADC_x_, ADC_y_, ADC_z_) using a 2-point fit to solve a mono exponential diffusion decay in Matlab (Mathworks, Natick, MA). A final trace apparent diffusion coefficient (ADC) map was calculated (ADC = [ADC_x_ + ADC_y_ + ADC_z_] / 3). ECV maps were calculated online using pre/post T1 maps derived from a standard motion-corrected T1 fitting technique [9] and collected hematocrit.

For quantitative regional detection and estimation of fibrosis burden, ADC and ECV maps were segmented into 6 American Heart Association (AHA) segments. Positive regions for myocardial fibrosis were defined as: mean ADC > 2.0 μm^2^/ms [12] and mean ECV > 30 % [9]. Fibrosis burden was defined as the number of positive segments over the total number of segments.

Two-sample t-tests were performed to test for significance between mean values of fibrotic and non-fibrotic regions for ADC and ECV. Significance was denoted as *p* < 0.05 and the calculations were performed in Matlab. To statistically test for agreement in regional detection, Cohen’s Kappa tests were performed along with calculating sensitivity, specificity, positive predictive value (PPV), and negative predictive value (NPV) using ECV as the gold-standard reference. A Bonferroni correction was performed manually to the significant difference testing of regional detection scores by lowering the significance limit to *p* < 0.002. For fibrosis burden, Bland-Altman analysis [23] and intra-class correlation (ICC) [24] was performed to test for correspondence and agreement.

## Results

**Fig. 1:**
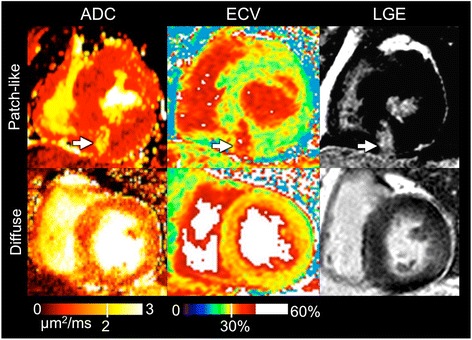
Representative examples of patch-like and diffuse representations of myocardial fibrosis in ADC, ECV, and LGE images. Although not used for quantitative analysis, LGE is provided for visual context. Regional patches of myocardial fibrosis (*white arrow*) are visualized as a hyperintense region in ADC, ECV, and LGE images. Diffuse presentation of myocardial fibrosis is qualitatively more conspicuous for both ADC and ECV image with “pepper-like” hyper intensity texture. Note that for the LGE image, appropriate window-leveling is required to properly visualize the same “pepper-like” hyper intensity

Qualitatively, all three ADC, ECV, and LGE were concordant in displaying patch-like presentation of myocardial fibrosis (Fig. 1). Patch-like presentation of myocardial fibrosis accounted for about 50 % (33/60) of the total number of positive fibrosis segments found on ECV. For diffuse presentations of myocardial fibrosis, ADC and ECV maps demonstrated qualitatively closer visual agreement. LGE required appropriate window-leveling to determine the presence of diffuse myocardial fibrosis, in which remote slices without hyperintensity must be identified (e.g. basal short-axis slices far from apical presentations of diffuse myocardial fibrosis).

**Fig. 2:**
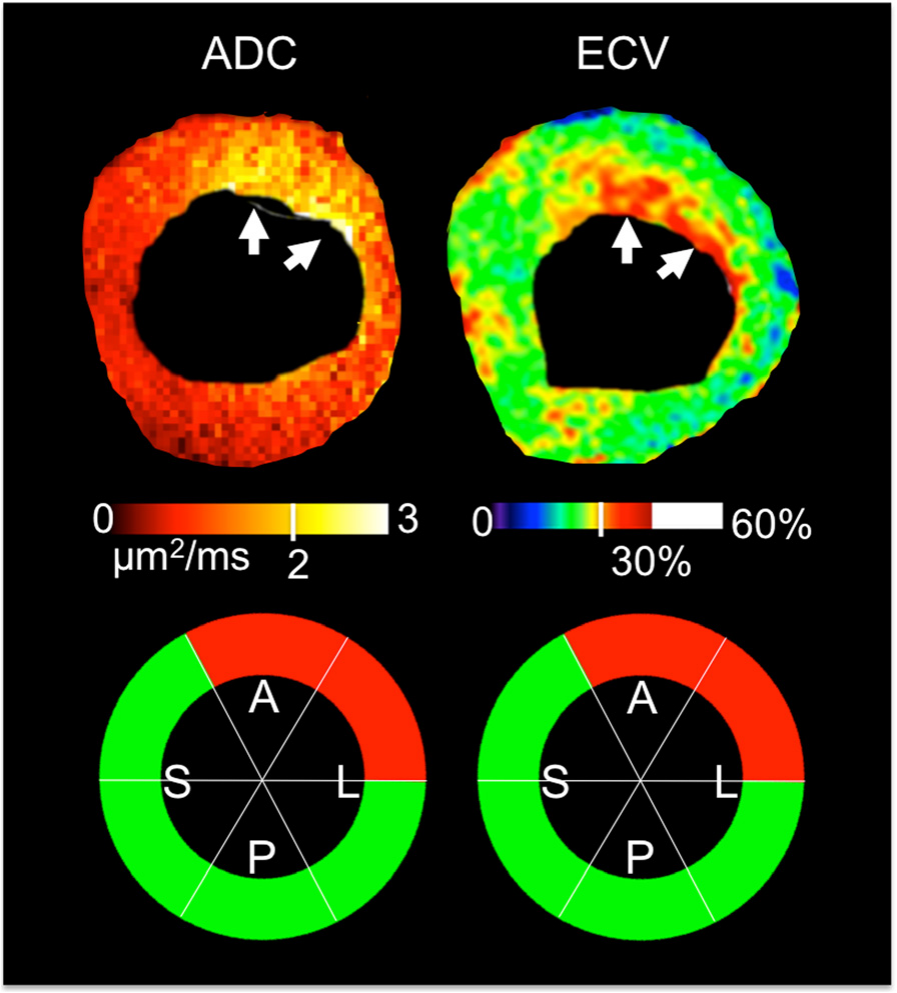
Representative example of processed ADC and ECV maps with associated AHA wheels including manual LV segmentation (*top row*) and AHA wheels (*bottom row*). Qualitatively, the ADC and ECV are in agreement with matching endocardial presentation of fibrosis in the anterior and anteriolateral AHA segments. This is further substantiated quantitatively with excellent agreement in the AHA wheels

**Fig. 3:**
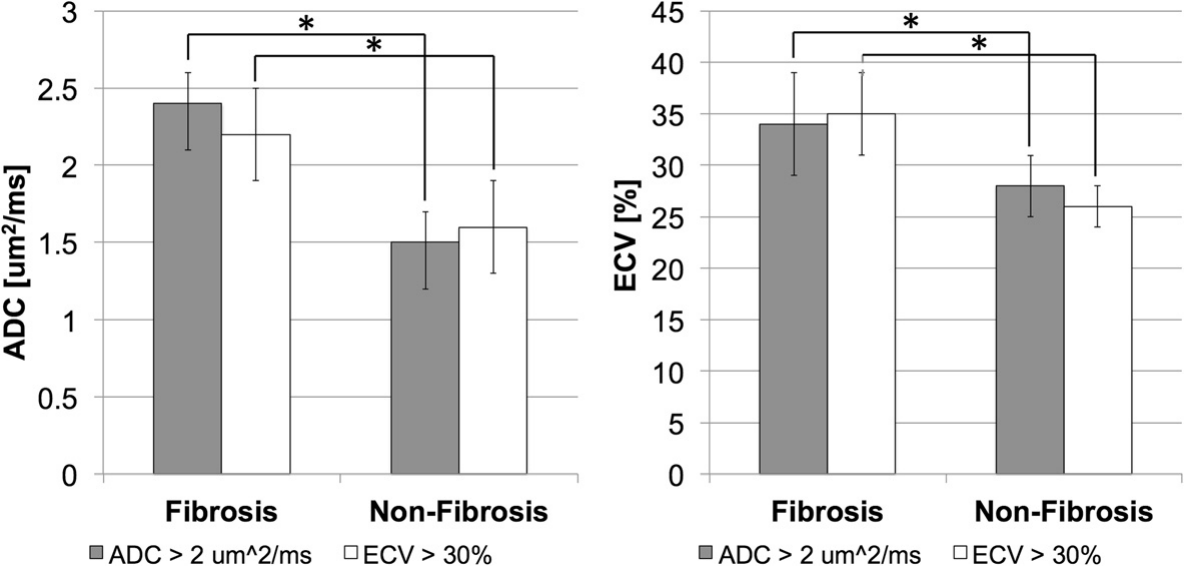
ADC and ECV in fibrosis and non-fibrosis regions defined by either ADC >2 μm^2^/ms or ECV > 30 % were compared. Both ADC and ECV were significantly (*p* < 0.01) higher in fibrosis than non-fibrosis regions for both criteria

**Fig. 4:**
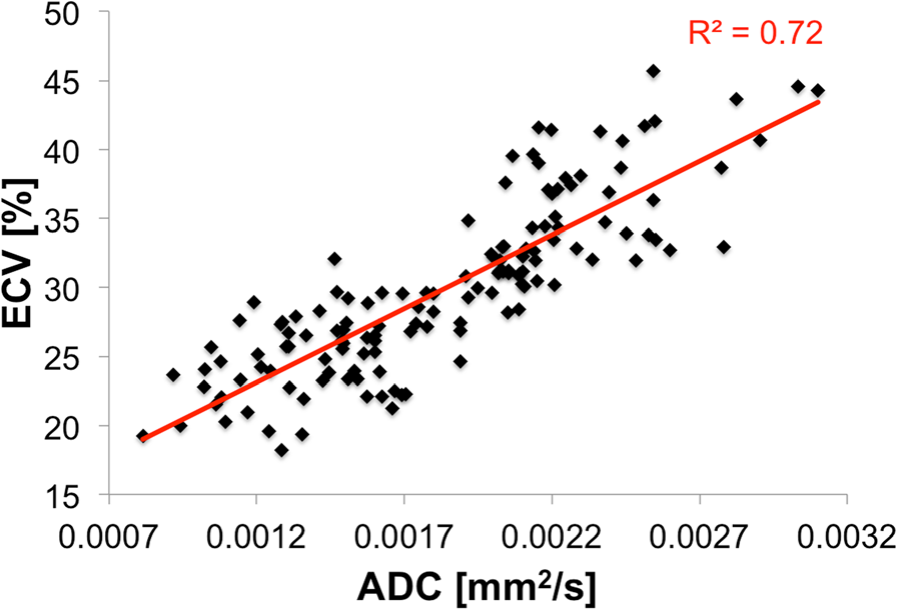
Correlation between mean ADC and ECV of the 138 AHA segments was substantial (R^2^ = 0.72). ADC and ECV ranged from 0.7 to 2.9 μm^2^/ms and 16 to 46 %, respectively

**Fig. 5:**
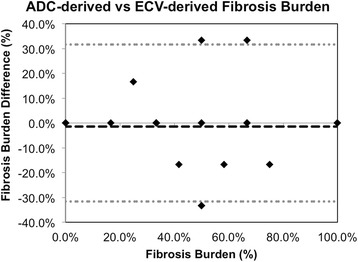
Bland-Altman plot of ADC-derived fibrosis burden compared with ECV-derived fibrosis burden. Qualitatively, no systematic bias errors were observed. The ICC demonstrated strong agreement (0.85) and mean bias was minimal (1.4 %)

Quantitatively, ADC (2.4 ± 0.2 μm^2^/ms) of fibrotic regions (ADC > 2.0 μm^2^/ms) was significantly (*p* < 0.01) higher than ADC (1.5 ± 0.2 μm^2^/ms) of non-fibrotic regions (Figs. 2 and 3). Similarly, ECV (35 ± 4 %) of fibrotic regions (ECV > 30 %) was significantly (*p* < 0.01) higher than ECV (26 ± 2 %) of non-fibrotic regions. In fibrotic regions defined by ECV, ADC (2.2 ± 0.3 μm^2^/ms) was again significantly (*p* < 0.05) higher than ADC (1.6 ± 0.3 μm^2^/ms) of non-fibrotic regions. In fibrotic regions defined by ADC criterion, ECV (34 ± 5 %) was significantly (*p* < 0.01) higher than ECV (28 ± 3 %) in non-fibrotic regions. Excellent inter-class (Pearson) correlation (R^2^ = 0.72) between ECV and ADC was observed (Fig. 4). ADC-derived and ECV-derived fibrosis burdens were in substantial agreement (ICC = 0.83) and qualitatively did not yield any systematic biases (mean bias = 1.4 %) (Fig. 5).

**Fig. 6:**
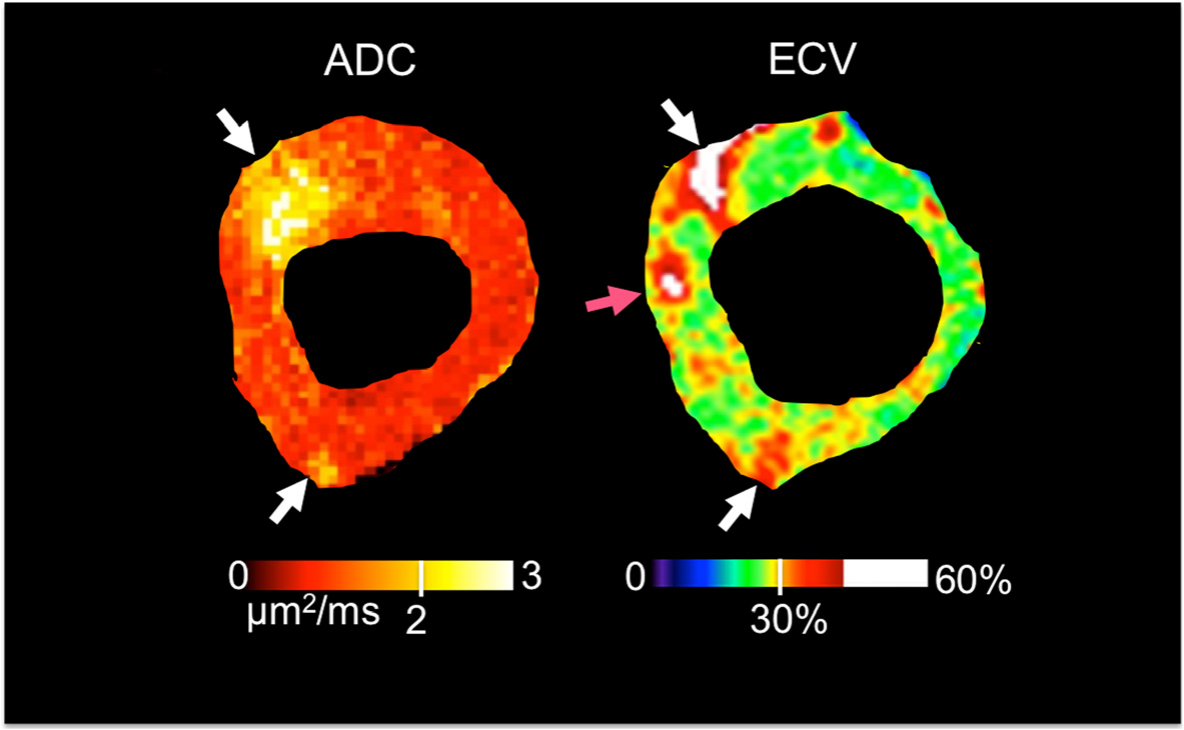
Representative example of a discordant segment (anteroseptal) between ADC and ECV. Note that the ADC map was acquired in systole, while ECV was acquired during diastole. Concordant segments (*white arrows*) are found in the anterior and posterior regions demonstrating hyper intense regions in both ADC and ECV. The hyperintense region (*pink arrow*) detected in the anteroseptal segment of the ECV map is absent in the ADC map resulting in a discordant AHA segment

Regional detection (Table 3) between ADC and ECV of diffuse fibrosis yielded substantial agreement (κ = 0.66) with high sensitivity, specificity, PPV, NPV, and accuracy (0.80, 0.85, 0.81, 0.85, and 0.83, respectively). About 83 % (115/138) of the number of segments was in agreement. A vast majority (18/23 = 78 %) of the discordant segments included detection of large fibrotic regions that straddled the border between two segmental zones (e.g. anterolateral and anterior segments). The other 5 discordant segments (Fig. 6) demonstrated unique differences between ECV and ADC with hyperintense regions being present in one and completely absent in the other.

**Table 3:** Fibrosis Detection ADC vs ECV

		ECV	
		+	-
ADC	+	49	12
	-	11	66
# of segments in agreement		115 (83 %)	
Cohen’s Kappa (κ)		0.66*	
Paired t-test test (*p*)		NS	
# of ADC fibrosis segments		60 (44 %)	
# of ECV fibrosis segments		61 (44 %)	
Sensitivity^a^		0.80	
Specificity^a^		0.85	
PPV^a^		0.81	
NPV^a^		0.85	

*NS* not significant

*p < 0.001

^a^ECV was gold standard

## Discussion

ADC was capable of detecting both patch-like and diffuse presentation of myocardial fibrosis agreeing closely with ECV. ADC was significantly higher in fibrotic defined by ECV (ECV >30 %) with ECV also being significantly higher in fibrotic regions defined by ADC (ADC > 2.0 μm^2^/ms). ADC was also strongly correlated (R^2^ = 0.72) with ECV yielding the possibility of quantifying the degree of fibrosis. Fibrosis burdens derived from ECV and ADC were substantially in agreement with minimal mean bias. Regional detection analysis demonstrated that ADC yielded substantial agreement with ECV with high sensitivity, specificity, PPV, NPV, and accuracy.

The vast majority (18/23 = 78 %) of differences between ECV and ADC in the regional fibrosis detection analysis were most likely due to cardiac phase mis-match since these discordant segments encompassed large (≥2 segments) fibrotic regions that were on the border of two or more segments. ECV and ADC maps acquired for these cases were in completely different cardiac phases (end diastole vs end systole). Although infrequent (5/138 = 4 %) in regard to the total number of segments analyzed, the other discordant segments (5/23 = 22 %) suggest an inherent difference between ECV and ADC. Several possibilities could account for these few differences including the presence of unaccounted non-fibrotic tissue that could affect ECV but not ADC, the presence of higher order motion that cannot be fully compensated affecting ADC but not ECV [12], or the potential regional heterogeneity related to proximity to the receiver coil affecting ADC more than ECV due to low signal-to-noise ratio [25]. Further investigation is needed to pinpoint the exact source of these potentially inherent differences between ADC and ECV.

Clinically, this study demonstrated the potential for DW-CMR as a contrast-free alternative to LGE and ECV for myocardial fibrosis detection. Extending previous work that identified DW-CMR’s ability to detect replacement fibrosis [12, 17, 18], the presented work demonstrated an additional sensitivity to diffuse presentations of myocardial fibrosis when compared to ECV and LGE. Although not rigorously tested in this study, our preliminary application of DW-CMR in HCM patients and previous study in chronic MI porcine [12] suggest that differentiating between diffuse and replacement fibrosis using DW-CMR is possible. DW-CMR is similar to ECV and LGE in its ability to differentiate between diffuse and replacement fibrosis by means of examining the qualitative presentation of a quantitatively observed elevated (>2 um^2^/ms) region. If the observed elevated region is focal in presentation and has a higher ADC value than other suspected elevated regions, then the observed region is more likely replacement fibrosis. However, further rigorous studies are needed to be more quantitative and exact in differentiating between replacement and diffuse fibrosis using DW-CMR. Additionally, DW-CMR has also demonstrated clinical potential in detecting edema in acute myocarditis [26].

Practically, DW-CMR requires more robustness in order for it to feasibly be an effective LGE or ECV contrast-free alternative used in a clinical setting. The DW-CMR technique in this study relied on manually finding the most quiescent period to trigger the motion compensated diffusion preparation. About half the patients required end systolic triggering due to high and unstable heart rates that greatly shortened the duration of end diastole and/or inconsistent triggering. Automatic or semi-automatic methods in determining the ideal cardiac phase to trigger would make this technique more feasibly push-button. Another major practical limitation of the DW-CMR approach used in this study was the low spatial coverage (4 slices at 8 mm thickness = 32 mm coverage) given the limited 5 min clinical scan time. In principle, this DW-CMR approach could achieve whole LV coverage (~80 mm) but would require at least 12.5 min of scan time. Specifically for HCM patients with larger LV masses, the minimum required scan time would need to be closer to 15 min to sufficiently cover the whole LV (~100 mm). As a result, this technical limitation restricted the overall study design, in which estimation of whole LV fibrosis burden could not be assessed. One possible future technical solution is the potential coupling of the motion compensated diffusion preparation with a time-efficient hybrid radial-Cartesian segmented 3D readout [27].

## Conclusion

DW-CMR is a contrast-free non-invasive quantitative technique that is sensitive to diffuse presentations of myocardial fibrosis in HCM patients. When compared to the established contrast-enhanced ECV-CMR, DW-CMR is able to yield comparable detection and characterization of myocardial fibrosis.

## Funding

This study was supported in part by the research grants of National Institute of Health National Institute of Biomedical Imaging and Bioengineering (1F31EB018152-01A1), National Natural Science Foundation of China (81370036 and 81130029), the Fundamental Research Funds for the Central Universities of China (3332013105), Capital Clinically Characteristic Applied Research Fund of China (Z151100004015141), and Beijing Natural Science Foundation (7152124).
